# Environmental Assessment and Evaluation of Oxidative Stress and Genotoxicity Biomarkers Related to Chronic Occupational Exposure to Benzene

**DOI:** 10.3390/ijerph16122240

**Published:** 2019-06-25

**Authors:** Isabele C. Costa-Amaral, Leandro V. B. Carvalho, Marcus Vinicius C. Santos, Daniel Valente, Angélica C. Pereira, Victor O. Figueiredo, Juliana Mendonça de Souza, Vinicio S. Castro, Maria de Fátima Trancoso, Antônio Sérgio A. Fonseca, Vanessa G. Milagres, Michele P. R. Mendes, Maria José N. Paiva, Leiliane C. André, Renato M. Borges, Marco Antônio C. Menezes, Sérgio R. Alves, Eline S. Gonçalves, Herbert Ary Sisenando, Jamila A. Perini, Mônica S. Oliveira, Maria Juliana Moura-Correa, Liliane R. Teixeira, Andrew R. Collins, Rita de Cássia O. C. Mattos, Paula N. Sarcinelli, Ariane L. Larentis

**Affiliations:** 1 Centro de Estudos da Saúde do Trabalhador e Ecologia Humana (CESTEH), Escola Nacional de Saúde Pública Sergio Arouca (ENSP), Fundação Oswaldo Cruz (Fiocruz), Rua Leopoldo Bulhões 1480, Manguinhos, Rio de Janeiro-RJ, CEP 21041-210, Brazil; leandro.carvalho@ensp.fiocruz.br (L.V.B.C.); m.vinicius_csantos@yahoo.com.br (M.V.C.S.); dvalentebio@gmail.com (D.V.); geli.cardoso@hotmail.com (A.C.P.); victorof30@hotmail.com (V.O.F.); jl.mendonsa@hotmail.com (J.M.d.S.); viniciodecastro@gmail.com (V.S.C.); fatimatroncoso@gmail.com (M.d.F.T.); asergio@ensp.fiocruz.br (A.S.A.F.); renatomarcullo@gmail.com (R.M.B.); elinesg@gmail.com (E.S.G.); marcocarneiromenezes@gmail.com (M.A.C.M.); sergio.rabello@fiocruz.br (S.R.A.); mjulianamc@gmail.com (M.J.M.-C.); lilianeteixeira@ensp.fiocruz.br (L.R.T.); mattos@ensp.fiocruz.br (R.d.C.O.C.M.); paulasarcinelli@hotmail.com (P.N.S.); 2 Departamento de Análises Clínicas e Toxicológicas, Faculdade de Farmácia, Universidade Federal de Minas Gerais (UFMG), Av. Presidente Antônio Carlos 6627, Pampulha, Belo Horizonte-MG, CEP 31270-901 Brazil; vaahgm@gmail.com (V.G.M.); micheleprm@yahoo.com.br (M.P.R.M.); mjnpaiva@yahoo.com.br (M.J.N.P.); leiliane@ufmg.br (L.C.A.); 3 Departamento de Análises Clínicas e Toxicológicas (DACT), Centro de Ciências da Saúde (CCS), Universidade Federal do Rio Grande do Norte (UFRN), Rua General Cordeiro de Faria S/N, Petrópolis, Natal-RN, CEP 59012-570, Brazil; herbertsisenando@yahoo.com.br; 4 Laboratório de Pesquisa de Ciências Farmacêuticas, Unidade de Farmácia, Centro Universitário Estadual da Zona Oeste (UEZO), Av. Manuel Caldeira de Alvarenga 1203, Campo Grande, Rio de Janeiro-RJ, CEP 23070-200, Brazil; jamilaperini@yahoo.com.br; 5 Laboratório de Biodosimetria, Departamento de Dosimetria (DIDOS), Instituto de Radioproteção e Dosimetria (IRD), Comissão Nacional de Energia Nuclear (CNEN), Av. Salvador Allende 3773, Recreio, Rio de Janeiro-RJ, CEP 22783-127, Brazil; m.stuck@yahoo.com.br; 6 Secretaria Municipal de Saúde/Prefeitura Municipal de Porto Alegre (SMS-POA), Av. João Pessoa 325, Cidade Baixa, Porto Alegre-RS, CEP 90040-001, Brazil; 7 Department of Nutrition, Institute of Basic Medical Sciences, University of Oslo (UiO), POB 1046, Blindern 0316 Oslo, Norway; a.r.collins@medisin.uio.no

**Keywords:** benzene, occupational and environmental health, chronic exposure, oxidative stress, genotoxicity and formamidopyrimidine DNA-glycosylase

## Abstract

Environmental and occupational exposure to benzene from fuels is a major cause for concern for national and international authorities, as benzene is a known carcinogen in humans and there is no safe limit for exposure to carcinogens. The objective of this study was to evaluate the genotoxic effects of chronic occupational exposure to benzene among two groups of workers: filling station workers (Group I) and security guards working at vehicles entrances (Group II), both on the same busy highway in Rio de Janeiro, Brazil. Sociodemographic data on the workers were evaluated; the concentration of benzene/toluene (B/T) in atmospheric air and individual *trans*,*trans*-muconic acid (*tt*MA) and *S*-phenylmercapturic acid (*S*-PMA) were measured; oxidative stress was analyzed by catalase (CAT), glutathione *S*-transferase (GST), superoxide dismutase (SOD), thiol groups (THIOL) and malondialdehyde (MDA); genotoxicity was measured by metaphases with chromosomal abnormalities (MCA) and nuclear abnormalities, comet assay using the enzyme formamidopyrimidine DNA glycosylase (C-FPG), and methylation of repetitive element LINE-1, *CDKN2B* and *KLF6* genes. Eighty-six workers participated: 51 from Group I and 35 from Group II. The B/T ratio was similar for both groups, but Group I had greater oscillation of benzene concentrations because of their work activities. No differences in *tt*MA and *S*-PMA, and no clinical changes were found between both groups, but linearity was observed between leukocyte count and *tt*MA; and 15% of workers had leukocyte counts less than 4.5 × 10^9^ cells L^−1^, demanding close worker’s attention. No differences were observed between the two groups for THIOL, MDA, MCA, or nuclear abnormalities. A multiple linear relationship was obtained for the biomarkers MCA and C-FPG. A significant correlation was found between length of time in current job and the biomarkers C-FPG, MCA, GST, and MDA. Although both populations had chronic exposure to benzene, the filling station workers were exposed to higher concentrations of benzene during their work activities, indicating an increased risk of DNA damage.

## 1. Introduction

Gasoline contains aromatic hydrocarbons like benzene, toluene, ethylbenzene, and xylenes (BTEX), among which benzene has the greatest toxicological risk because of its carcinogenic properties [1,2,3,4,5,6,7]. Although benzene is present in low concentrations in gasoline and other finished products, economic development policies that stimulate the consumption of petroleum-based fossil fuels means that exposure to this compound, especially because of its presence in gasoline, is of particular concern in the ambit of environmental and occupational health [2,8,9].

The population at large is also environmentally exposed to benzene and other hydrocarbons due to the volatilization of solvents present in gasoline both at filling stations—from storage tanks and while vehicles are filled—and from vehicle emissions, which leads to their widespread diffusion. These emissions are particularly significant for people who live in the vicinity of certain types of industrial plants and filling stations [10]. However, little research has been carried out to assess the genotoxicity biomarkers of benzene exposure and other organic compounds present in gasoline and other fuels in Brazilian filling stations [11,12,13,14,15,16,17,18,19,20]. Recently, a thematic dossier of the occupational health journal *Revista Brasileira de Saúde Ocupacional* (RBSO, Fundacentro) called “Occupational Exposure to Benzene in Filling Stations and Fuel Distribution Chain in Brazil” was published, which provides evidence of the continuous exposure of filling station workers to this compound [4].

The mechanism of action of the hematopoietic toxicity of benzene is largely unknown, both in terms of the development of peripheral cytopenia and in the induction of acute myeloid leukemia [21,22]. However, in simple terms, there are some well-defined key events that seem to be prerequisites for this chemical to become toxic. These key events are cited in the description of the mode of action of benzene, namely: (1) the metabolization of benzene to benzene oxide; (2) the interaction of this metabolite with crucial cells in bone marrow; (3) stimulation of bone marrow cells; (4) clonal proliferation of the initiated cells; and (5) the development of leukemia [21,22]. The critical stage described by Meek and Klaunig [22] includes additional oxidative damage of DNA and important cellular macromolecules, inducing mutations and the clonal proliferation of mutated cells. Evidence indicates that the metabolites of benzene may interfere in the cell cycle and may also induce the apoptosis of precursor cells in the hematopoietic system, while also altering important cell signaling pathways in this system, resulting in cytotoxicity [21,23]. However, multiple pathways are involved in the mode of action and leukemogenic process of benzene and its metabolites, making it hard to define through one single mode of action [24].

One important factor concerning benzene biotransformation is that humans metabolize this compound more efficiently when exposed to low concentrations, which suggests the involvement of two metabolic pathways, one of which can be saturated at higher concentrations, leading to a sharper leukemia risk curve at lower environmental exposure levels [25]. Recent studies have indicated a supralinear dose-response relationship for benzene metabolism at low doses (<1 ppm) by comparing the kinetic models of both metabolic benzene pathways, suggesting that the curve slope is steeper at low exposure doses [25,26,27,28]. This risk of leukemia at low doses could be related to the fact that benzene is metabolized more efficiently at low exposure concentrations, resulting in the increased production of its reactive metabolites, which are hematotoxic. As such, the production of these metabolites with toxic activity could lead to greater toxic effects than would be expected for individuals exposed to low concentrations of benzene in the air [25]. However, there is as yet no consensus in the literature on this, since the relationship between benzene exposure and relative risk has been described as a linear model [3].

The metabolism of benzene therefore plays an important role in its toxicity, although it is not yet clear which of its metabolites are responsible for its toxic effects [24]. These effects may be generated by: the creation of a covalent bond in important biomolecules, like proteins and enzymes (tubulin, histones, and topoisomerase II); the generation of oxidant species, resulting in oxidative stress; and damage to the DNA itself by DNA-binding proteins, cross-linking or single- or double-strand breakage, and chromosome aberrations, especially in chromosomes 5 and 7, which are affected in the development of acute myeloid leukemia. As such, the relationship between the genotoxic potential of benzene metabolites and their carcinogenic effects still needs to be more clearly elucidated [21,29,30,31].

In view of the genotoxic effects of benzene metabolites related to potential illness risks at low exposure doses, there is a need to understand chronically exposed populations. In addition, it is important to consider that benzene arising from the combustion of vehicle gasoline generates significant environmental exposure, making it impossible to have an effectively non-exposed population, mainly in large urban centers. In this context, this descriptive, exploratory, cross-sectional study was carried out in 2014 and 2015 in Rio de Janeiro, Brazil, with the aim of comparing measurements for evaluating environmental exposure to benzene and toluene and their metabolites, as well as clinical parameters and the biomarkers oxidative stress and genotoxicity, between two groups of workers with occupational exposure to low concentrations of these substances, but one of whose activities brings them into direct contact with a benzene source.

## 2. Materials and Methods

**Figure 1:**
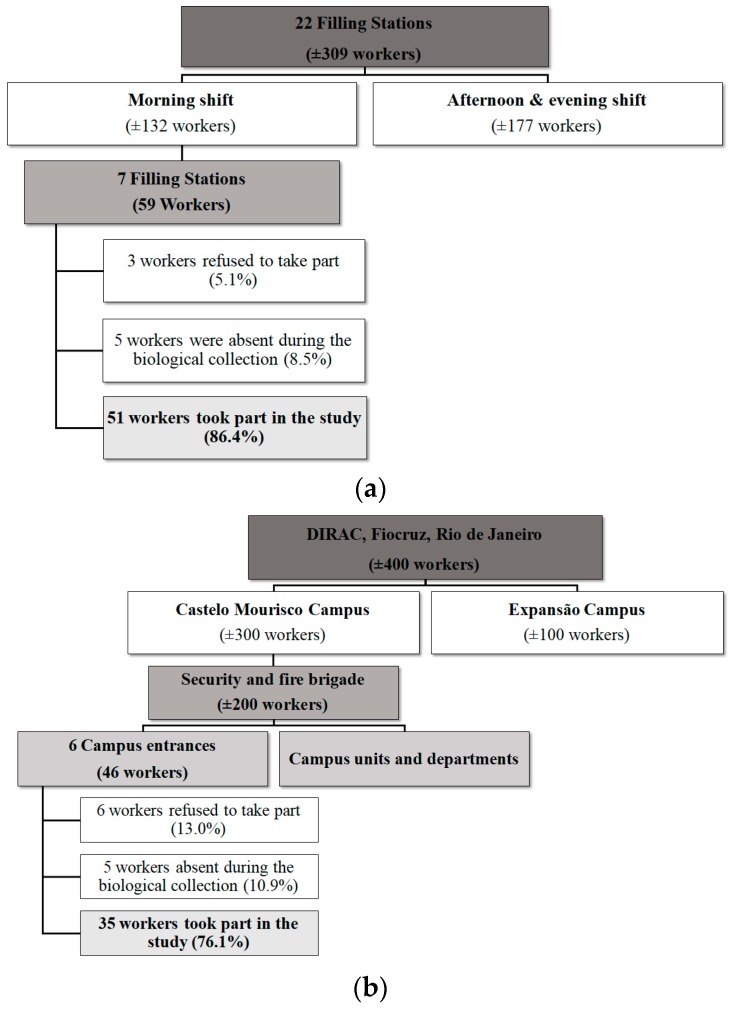
Diagrams of the selection of the two groups of workers exposed to benzene. (**a**) Group I: Workers of Filling Stations; (**b**) Group II: Workers of Campus Entrances.

The study population consisted of 86 workers (≥18 years) with environmental and occupational exposure to low concentrations of benzene, who were divided into two groups. The first group (Group I) was made up of 51 employees of filling stations in the west zone of Rio de Janeiro, and the second group (Group II) consisted of 35 security guards who worked at the road entrances to the Castelo Mourisco campus of Fiocruz in the north zone of the same city. All the workers were selected for having fixed workplaces (filling stations or campus entrances). The workplaces of both groups of workers were situated in different parts of the same busy, multi-lane highway that carries an intense flow of cars, buses, and trucks into and out of the city. This means that both groups of workers had background environmental exposure to benzene at their workplaces due to vehicle emissions. On this same highway there are also steelworks and oil refineries, again contributing to environmental benzene exposure. The difference between the benzene exposure of the two groups was therefore due to their jobs. Members of Group I dispensed gasoline, which puts them in direct contact with a benzene source, while members of Group II did not suffer this occupational exposure. Owing to the significant distance between the Group I workplaces and the biological sample processing site, only morning shift workers were assessed, thus ensuring identical sample processing for both groups of workers. Figure 1 provides a diagrammatic depiction of how the workers from the two groups were selected.

The data on the workers were gathered in standardized questionnaires [32], from their medical history, and from laboratory (hematological and toxicological) exams. In order to analyze their work activities, the following sociodemographic and occupational variables were used: age (divided into age groups), sex, race/ethnicity, marital status, education level, job, work activities, and length of time in current job. Sex, age, tobacco use, and alcohol use were used as control variables in the correlations. “Tobacco use” was divided into three categories: never smoked, used to smoke, and smokers; “use of alcohol” was divided into never drunk, used to drink, and drinks. All subjects gave their informed consent before they were included in the study. The study was conducted in accordance with the Declaration of Helsinki, and the protocol was approved by the Ethics Committee of ENSP/Fiocruz (CEP/CAAE 17438013.5.0000.5240 and 38643014.7.0000.5240).

### 2.1. Environmental Assessment

The environmental assessment of the workplaces was conducted by quantifying benzene and toluene in atmospheric air using a gas chromatograph with a flame ionization detector (Focus, Thermo-Finnigan, Waltham, MA, EUA), as described in method 1501 of the *NIOSH Manual of Analytical Methods* [33]. The capillary column used was a Carbowax 20 M (Agilent, Santa Clara, CA, EUA) with 60 m length, 0.32 mm internal diameter, and 0.25 μm film thickness. The limits of quantification encountered were 5.60 μg m^−3^ for benzene and 6.70 μg m^−3^ for toluene. The assessment was undertaken using active samplers at the workplaces (filling stations and campus entrances) of the two groups under study. The air samples were collected in activated carbon tubes (Anasorb CSC, SKC, Eighty Four, PA, USA) with 50 and 100 mg sorbent) using air sampling pumps (PCXR4, SKC) calibrated for a flow rate of 0.2 L min^−1^. The sampling points were located near the areas where the workers circulated and the air was collected over a 150-minute period. At the filling stations, the sampling points were set at 1.5 m above ground level where the workers circulated, near the fuel pumps.

### 2.2. Clinical Assessment

The data were obtained from clinical evaluations made of all the workers, which were done by an occupational physician at their respective workplaces using a clinical questionnaire as a script. Hematology tests were done at the Germano Sinval Faria School Health Center/ENSP at Fiocruz.

### 2.3. Evaluation of Exposure, Oxidative Stress, and Genotoxicity Biomarkers

The evaluation was conducted by analyzing biomarkers of exposure *trans*,*trans*-muconic acid (*tt*MA) and *S*-phenylmercapturic acid (*S*-PMA), biomarkers of oxidative stress catalase (CAT), superoxide dismutase (SOD), glutathione *S*-transferase (GST), thiol groups (THIOL), and malondialdehyde (MDA), and biomarkers of genotoxicity (metaphases with chromosomal aberrations (MCA), number of chromosome breaks (NCBk), fragments (Frag), premature chromatid separation (PCS), micronuclei (MN), binucleated cells (BNCs), broken egg cells (BECs), and comet assay with FPG (C-FPG). Blood and urine samples were collected at the workplaces in the morning, stored in thermal boxes containing artificial ice, and transported to the laboratory. All laboratory processing procedures were performed in the afternoon of the same day.

The urine samples used to assess the biomarkers of exposure (*tt*MA and *S*-PMA) were collected at the end of the working day in polyethylene pots, fractionated, and stored in an ultra-freezer (−80 °C) until analysis. Urine samples were used for *S*-PMA measurement after three years’ storage. Waidyanatha et al. [34] have used samples after eight years’ storage with satisfactory results.

The blood samples used to assess the biomarkers of effect were collected in vacutainer tubes with different types of anticoagulants (three with sodium heparin, one with EDTA, and one without anticoagulant). The tubes with EDTA were used for the hematology tests. The samples in the tubes with sodium heparin were immediately analyzed for biomarkers of genotoxicity. The tube without anticoagulant was used for the analyses of biomarkers of oxidative stress, using UV-visible spectrophotometry (UV-1601, Shimadzu, Kyoto, Japan) with serum as a matrix. For this procedure, the tube without the anticoagulant was centrifuged at 1600 × *g* for 10 min and the serum obtained was separated into individual aliquots for each analysis. These aliquots were stored at −80 °C in an ultra-freezer until analysis. Aliquots of 250 µL whole blood from the tube with sodium heparin were transferred to 1.5 mL microtubes and stored in an ultra-freezer (−80 °C). They were then sent to the Department of Nutrition of the University of Oslo, where the comet assays with FPG were conducted. The urine aliquots for analysis of *S*-PMA were sent to the Department of Clinical and Toxicological Analyses at UFMG, where they were analyzed. Methylation analyses were done at the Laboratory of Molecular Carcinogenesis at INCA. The other biomarker analyses were conducted at the Toxicology Laboratory/CESTEH/ENSP at Fiocruz.

#### 2.3.1. Determination of *trans*,*trans*-Muconic Acid (*tt*MA)

*tt*MA was determined by high-performance liquid chromatography with UV-visible detection using a C18 column (250 mm × 4.6 mm × 5 μm) and UV detection at 264 nm, as described by Ducos et al. [35] and modified by Paula et al. [36]. Before the HPLC analysis, the urine samples were put through solid-phase extraction with quaternary amine resin (SAX—strong anion exchange). Next, they were submitted to chromatographic analysis under the following conditions: mobile phase methanol/acetic acid 1% (10:90); flow rate of 1 mL min^−1^; sample volume of 20 µL; oven temperature of 40 °C. The concentration of the samples was calculated in mg L^−1^ by linear regression and the results were corrected by creatinine concentration. The detection limit of the method was 0.11 mg L^−1^.

#### 2.3.2. Determination of *S*-phenylmercapturic Acid (*S*-PMA)

The method used is called extraction with low-temperature liquid-liquid partitioning and is described by Gomes et al. [37] (with modifications). Briefly, the method consisted of extracting the urinary *S*-PMA from 600 µL urine (whose pH had been corrected to 1 with HCl 25%). This volume was added to a 2 mL Eppendorf tube. This sample was then centrifuged to precipitate the proteins and other compounds present in the urine. After this, 600 µL acetonitrile was added and the sample was agitated in a vortex mixer for 1 min. then centrifuged again under the same conditions as previously. The samples were then left overnight at −20 °C for partitioning to occur. The next day, 230 µL of the supernatant was collected, which was evaporated to dryness at approximately 80 °C. Next, the residue was resuspended in 50 µL BSTFA in the test tube. The sample was then agitated in a vortex mixer for 1 min, left in the ultrasound for 1 min, then agitated again for 15 s. For derivatization, the sample was transferred to a vial with an insert, which was put in a conventional microwave oven for 1 min at 20% power. Finally, using a manual injection syringe, 1 µL of the derivatized sample was injected into the gas chromatograph coupled to a quadrupole mass spectrometer (Agilent Technologies^®^). The limit of detection for the method was 0.95 µg L^−1^.

#### 2.3.3. Determination of Catalase Activity

Catalase activity in human serum was determined using the method described by Góth [38], in which enzyme activity was evaluated by hydrogen peroxide (H_2_O_2_) decomposition, measured at 405 nm. The reaction medium consisted of 500 μL H_2_O_2_ (65 mmol L^−1^) prepared in a sodium potassium phosphate buffer (0.06 mol L^−1^ and pH = 7.4) with 100 μL serum sample. This medium was incubated at 37 °C for 1 min. The reaction was then stopped with 500 μL ammonium molybdate (40 g L^−1^).

#### 2.3.4. Determination of Glutathione *S*-transferase (GST) Enzyme Activity

GST activity in serum was determined using the method originally described by Habig et al. [39], as adapted by Habdous et al. [40], which involves determining enzyme activity by the formation of the reaction product GS-DNB, monitored at 340 nm, using 1-chloro-2,4-dinitrobenzene (CDNB) as the substrate and reduced glutathione (GSH) as the co-substrate. The reaction medium consisted of 700 µL potassium phosphate buffer (0.1 mol L^−1^, pH = 5.5), 100 µL CDNB 25 mmol L^−1^, 100 µL GSH 50 mmol L^−1^ and 100 µL sample.

#### 2.3.5. Determination of Superoxide Dismutase (SOD) Enzyme Activity

The SOD assay kit used was manufactured by Cayman Chemical Company (Ann Arbor, MI, USA). It uses a tetrazolium salt for detection of superoxide radicals generated by xanthine oxidase and hypoxanthine. One unit of SOD is defined as the amount of enzyme needed to exhibit 50% dismutation of the superoxide radical. The reaction medium consisted of 200 μL radical detector and10 μL serum sample (dilution 1:5). The reaction was initiated with 20 μL xanthine oxidase. A calibration curve was prepared with an SOD standard and the samples and standards were read at 450 nm. The detection limit was 0.01 U mL^−1^.

#### 2.3.6. Thiol Group (THIOL) Analysis

The concentration of total thiols in serum was analyzed using the method described by Hu [41], which consists of quantifying the product of the reaction of thiol groups (-SH) and 5,5-dithiobis(2-nitrobenzoic acid), DTNB, measured at 412 nm. The reaction medium was composed of Tris-Cl buffer 0.25 mol L^−1^, EDTA 0.02 mol L^−1^, DTNB 0.01 mol L^−1^ and serum sample. A calibration curve was prepared with reduced glutathione (GSH) as the thiol standard. The concentration of thiol groups in the samples was ascertained by linear regression. The limit of detection of the method was 0.18 mmol L^−1^.

#### 2.3.7. Determination of Malondialdehyde (MDA)

The analysis of MDA, a product of lipid peroxidation, was done using a commercial kit produced by Cayman Chemical Company, which is based on the principle of quantifying the product from the reaction of MDA and thiobarbituric acid (TBA), measured at 532 nm. The reaction medium consisted of 1000 µL TBA, 25 µL sodium dodecyl sulfate and 25 µL serum sample. A calibration curve was prepared with the MDA standard. The concentration of MDA in the samples was calculated by linear regression. The limit of detection of the method was 0.47 µmol L^−1^.

#### 2.3.8. Chromosomal Anomalies (Chromosomal Aberrations, Breaks, Fragments, and Premature Chromatid Separation)

The test was done on a cell culture of peripheral blood lymphocytes. Cell division of the lymphocytes in culture was stimulated by phytohemagglutinin, as described (with adaptations) by Moorhead et al. [42]. The test consisted of preparing a cell culture obtained from 0.5 mL aliquots of whole human peripheral blood to which 5 mL RPMI medium, already enriched with phytohemagglutinin and fetal bovine serum, was added. Next, the lymphocytes were incubated at 37 °C for 48 h, after which cell division was halted at the metaphase stage by the addition of colchicine for 2 h. After incubation, the cells were treated with hypotonic solution and fixer to ensure a clear suspension containing only metaphase lymphocytes. To view the metaphases, six slides were prepared for each sample and assessed using an optical microscope with the Metafer^®^ 4 system. The metaphases were quantified and the frequency of alterations was analyzed. The aim was to observe at least 100 metaphase cells per individual in order to assure representativeness, as recommended by Carrano and Natarajan [43]. However, this number of cells was not observed for all the individuals assessed. The low mitotic index observed for some of the samples may be down to different possible causes or combinations of causes. These include the complexity and number of processes involved in obtaining the slides, from blood sampling to the slide preparation itself; the refinement software used to search for metaphases, which could have discarded valid cells; and the potential effect of cumulative exposure in the blood cell system, despite not being described, since it is well known that BTEX affects bone marrow blood cell production.

The results of the chromosomal alteration test were classified into four types of variables: metaphases displaying chromosomal aberrations (MCA), comprising the fraction of metaphase cells presenting at least one chromatid or chromosome aberration; number of chromosome breaks (NCBk) per metaphase, representing the observed number of chromosome and chromatid breaks; fragments (Frag), corresponding to the observed number of fragments; and metaphases presenting premature chromatid separation (PCS). The results were expressed as the number of aforementioned abnormalities per 100 metaphases. This data description allows the group to present all four analyzed abnormalities with a common basis, disregarding samples in which 100 metaphases were not observed.

#### 2.3.9. Nuclear Abnormalities (Micronuclei, Binucleated Cells and Broken Egg Cells)

This test was done by collecting exfoliated buccal mucosal cells using a cytobrush. The cells were transferred to a tube containing 5 mL NaCl 0.9%. They were then rinsed twice with the same saline solution and 100 µL aliquots of cell suspension were used to prepare the slides—four for each individual. All the slides were immersed in analytical grade ethanol for fixation and then left at ambient temperature to dry. The samples were then stained with Giemsa’s solution and stored under refrigeration until analysis. Two slides and 2000 exfoliated cells (1000 per slide) were observed per individual using a binocular optical microscope with 40× and 100× magnification. The nuclear abnormalities observed and classified by the test were: micronuclei (MN), binucleated cells (BNC), and broken egg cells (BEC). MN indicated a small nucleus contained in nucleated cells with a round or oval shape and a diameter varying between 1/3 and 1/16 of the size of the main nucleus and similar stain intensity to that of the original nucleus. BNCs were cells that contained two main nuclei of the same size and similar stain intensity. BECs were those cells where there was a constriction of the main nucleus, forming a bud attached to the main nucleus, with a diameter of 1/4 to 1/2 that of the main nucleus and similar stain intensity [44].The results were expressed by the frequency of MN, BNC, and BEC per 1000 cells counted.

#### 2.3.10. Comet Assay with FPG (C-FPG)

To isolate leukocytes from frozen whole blood, a 250 μL aliquot was thawed; 5 μL was mixed with 45 μL of PBS in a microcentrifuge tube, and centrifuged for 7 min at 700 × *g*, 4 °C. The supernatant was discarded and the pellet suspended in 45 μl of PBS and centrifugation was repeated using the same conditions. This process was repeated on more time and the pelleted leukocytes were used for the comet assay. Leukocytes were resuspended in 125 μL of 0.8% LMP agarose. Gels of 5 μL were set on NMP-precoated slides using a version of the comet assay with 12 mini-gels on each slide [45]. For the measurement of oxidized purines, after the lysis step, parallel slides were incubated for 30 min at 37 °C in either buffer (40 mmol·L^−1^ HEPES, 0.1 mol·L^−1^ KCl, 0.5 mmol·L^−1^ EDTA, 0.2 mgmL^−1^ BSA, pH = 8.0) or buffer with FPG. All the slides were incubated in alkaline solution (0.3 mol L^−1^ NaOH, 1 mmol L^−1^ EDTA) and submitted to electrophoresis at 0.8 V·cm^−1^ for 20 min. Slides were stained using Sybr Gold and comets were scored with Comet Assay IV software (Perceptive Instruments, England and Wales, UK). The results were expressed as DNA tail intensity.

#### 2.3.11. Methylation Analysis

The methylation of Kruppel-like factor 6 gene (*KLF6*), the hypermethylation of cyclin-dependent kinase inhibitor 2B gene (*CDKN2B*), and the hypomethylation of long interspersed nuclear element-1 (LINE-1) were evaluated using bisulfite treatment, biotin-labeled primers, and pyrosequencing, according to Santos [46]. These genes are known to be altered by benzene exposure [47,48].

### 2.4. Statistical Analysis

The descriptive analysis was conducted for the continuous data, which were presented as means, standard deviations, frequencies, and percentage distributions. The normality of distribution of the continuous variables was tested using the Kolmogorov-Smirnov test, with a 5% significance level. To compare the variables with normal distribution, Student’s *t*-test or ANOVA were used, while the Mann-Whitney or Kruskal-Wallis test were used for variables with non-normal distribution. The Pearson and Spearman correlation tests were run for the continuous variables with normal and non-normal distribution, respectively. Statistical significance was set at *p* < 0.05. Sex, age, tobacco use, and alcohol use served as control variables. Linear regression tests were conducted between the biomarkers of exposure (*tt*MA, *S*-PMA) and the biomarkers of oxidative stress and genotoxicity. All the data were analyzed with the aid of SPSS for Windows version 20.0 (Armonk, NY, EUA), and R^®^ version 3.5 (The R Foundation, Vienna, Austria).

## 3. Results

This study evaluated 86 workers with occupational and environmental exposure to benzene, with 51 in Group I and 35 in Group II. The sociodemographic characteristics and risk factors (tobacco use and alcohol use) of the two groups are presented in Table 1. Group 1 (filling station workers) was composed of 84.3% men and 15.7% women. Their mean age was 35 ± 11 years: 37 ± 11 years for the men and 29±7 years for the women. Most of the members of this group reported using alcohol (70.6%; *n* = 36) and not smoking (62.0%, *n* = 31). According to their own accounts, 72.5% (*n* = 37) worked as filling station attendants, 13.7% (*n* = 7) held management positions, 5.9% (*n* = 3) provided general maintenance services, 5.9% (*n* = 3) were lube technicians, and 2.0% (*n* = 1) were sales staff in the convenience store presented in the filling stations. The mean time in their current job was 7 ± 9 years. None of them reported using any personal or collective protective equipment during their work, during deliveries of fuel, or while doing fuel quality testing, which are activities that involve higher exposure rates. Group II (campus entrance workers) was composed of 82.9% men and 17.1% women. Their mean age was 43 ± 9 years: 44 ± 9 years for the men and 39 ± 9 years for the women. Most of the members of this group said they were non-smokers (57.1%) and half reported consuming alcoholic beverages (50.0%). As for their jobs, 96.6% (*n* = 28) were security guards and 3.4% (*n* = 1) were doormen. The mean time in their current positions was 15 ± 3 years, which meant there was no statistical difference between this group and Group I.

The atmospheric concentrations of benzene and toluene determined at the Group I and Group II workplaces are shown in Table 2. The Mann-Whitney test identified differences between the mean and median concentrations of benzene at the two workplaces; only the median was found to be different for the toluene concentration. The B/T emission ratios were 0.74 for Group I workplaces and 0.23 for Group II workplaces. No statistical difference was found between the two groups in the evaluation of exposure by means of the biomarkers *tt*MA and *S*-PMA (Table 2).

The results for *tt*MA and *S*-PMA did not present normal distribution, and the log transformation of *tt*MA (Ln*tt*MA) could be used for the analyses. Furthermore, no statistically significant correlation was found between *tt*MA and *S*-PMA. The results for *tt*MA and *S*-PMA for the different tobacco use categories are presented in Table 3. When the results for *tt*MA for the different tobacco use categories were compared, a statistical difference (*p* = 0.034) was found between the categories “never smoked” (0.19 ± 0.19) and “smokers” (0.45 ± 0.26), but only for Group I (Table 3). No difference was observed for *S*-PMA. The same comparison was done for the different categories of alcohol use and no difference was encountered.

**Figure 2:**
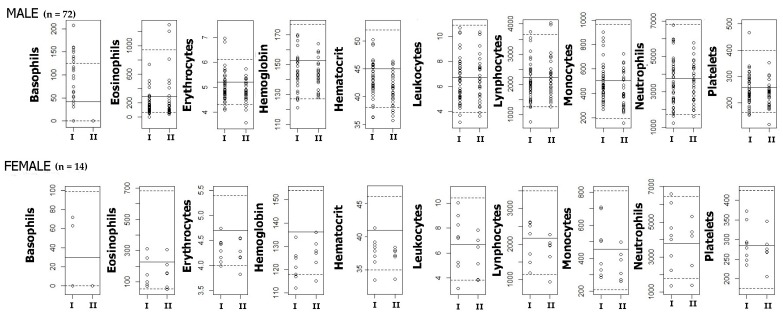
Results of hematotoxicity in the two groups of workers exposed to benzene, separated by sex. Mean and reference range for hematology laboratory counts: Basophilis (×10^6^ L^−1^): women 29.6 (0–99) and men 41.7 (0–125); Eosinophilis (×10^6^ L^−1^): women 228.5 (56–682) and men 284.6 (65–940); Erythrocytes (× 10^12^ L^−1^):women 4.7 (4.0–5.4) and men 5.2 (4.3–6.1); Hematocrit (L L^−1^): women 0.41 (0.35–0.46) and men 0.45 (0.38–0.52); Hemoglobin (g L^−1^): women 136.2 (118–154) and men 152.8 (127–177); Leukocytes (×10^9^ L^−1^): women 6.7 (3.84–10.4) and men 6.7 (3.9–10.9); Lynphocytes (×10^6^ L^−1^): women 2175.3 (1157–3500) and men 2223.2 (1265–3648); Monocytes (×10^6^ L^−1^): women 455.1 (208–807) and men 503.2 (192–968); Neutrophils (×10^6^ L^−1^): women 3777.3 (1804–6460) and men 3762.7 (1728–6820); Platelets (×10^9^ L^−1^): 284.1 (175–421) and men 258.6 (163–399) [50].

**Figure 3:**
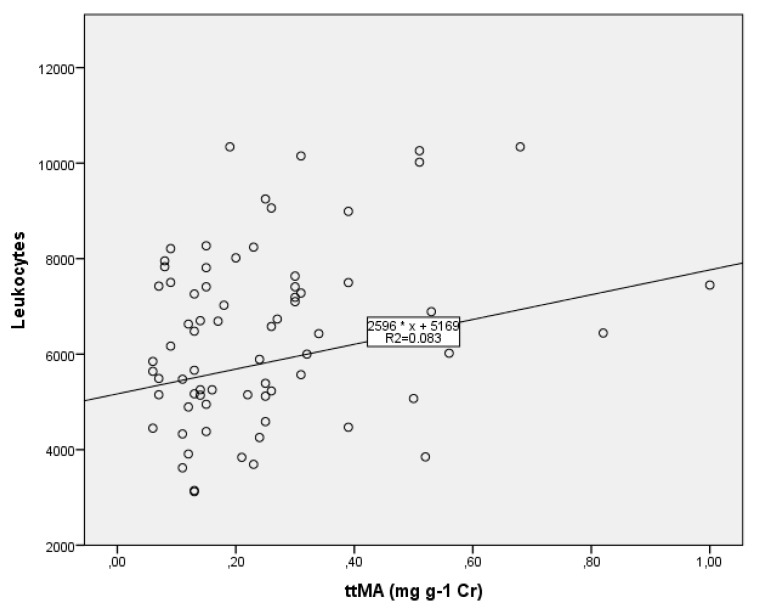
Plot of linearity between leukocyte count and *tt*MA. Linear relationship [F(2,66) = 2.970, *p* = 0.058, R^2^ = 0.083] between leukocyte count and *tt*MA (β = 5169, *p* = 0.031), using age as the control variable.

No difference between the two groups was found for the hematology tests (Figure 2). Even so, 15% (*n* = 13) of all the workers in this study presented a leukocyte count slightly below 4.5 × 10^9^ cells/L, the concentration proposed by our research group as warranting the worker’s attention and calling for close medical follow-up [49]. Positive Pearson correlations were found between Ln*tt*MA and the clinical data “total leukocyte count” (R = +0.275, *p* = 0.020) and “segmented neutrophils” (R = +0.272, *p* = 0.022). However, these correlations disappeared once they were controlled by the variables age, sex, alcohol and tobacco use. No correlation was found between *S*-PMA and the clinical data. The leukocyte count showed a significant linear relationship [F(2,66) = 2.970, *p* = 0.058, R^2^ = 0.083] with the predictor variables *tt*MA (ß = 5169, *t* = 2.204, *p* = 0.031) and using age as a control variable. The plot of linearity is shown in Figure 3. When sex, alcohol use, tobacco use, and duration of exposure were used as controls, the linear relationship was not significant. No linear relationship was observed when *tt*MA was replaced by *S*-PMA.

The results of the analyses of the biomarkers of oxidative stress and genotoxicity are presented in Table 4. The THIOL and MDA results for the two groups were statistically different. When the results of the oxidative stress biomarkers were compared across the categories of tobacco use and alcohol use, no differences between the groups were found. The Spearman rank correlation yielded negative correlations between GST activity and CAT (R = −0.387, *p* = 0.0001) and SOD activity (R = −0.390, *p* = 0.0001) and positive correlations between GST activity and MDA (R = +0.369, *p* = 0.001). It also yielded a negative correlation between SOD activity and MDA (R = −0.226, *p* = 0.046). However, when the data were corrected by age, sex, tobacco and alcohol use, correlations between GST activity and *tt*MA concentration (R = +0.422, *p* = 0.005) and between SOD activity and THIOL (R = +0.399, *p* = 0.008) were observed. As for the hematological data, CAT was found to correlate positively (by Spearman) with the number of basophils (R = +0.208, *p* = 0.054) and segmented neutrophils (R = +0.218, *p* = 0.044). Correlations were also found between GST and neutrophils (R = +0.512, *p* = 0.0001); between THIOL and eosinophils (R = +0.225, *p* = 0.037) and hemoglobin (R = +0.232, *p* = 0.032); and between MDA and neutrophils (R = +0.607, *p* = 0.0001), basophils (R = +0.366, *p* = 0.001), monocytes (R = +0.220, *p* = 0.005), and segmented neutrophils (R = −0.265, *p* = 0.018). However, when the analyses were controlled by sex, age, tobacco and alcohol use, correlations between CAT and red blood cells (RBCs; R = +0.524, *p* = 0.0001), basophils (R = +0.426, *p* = 0.004), and platelets (R = +0.425, *p* = 0.004); between GST and basophils (R = +0.506, *p* = 0.001); and between THIOL and eosinophils (R = +0.406, *p* = 0.007) were observed.

**Figure 4:**
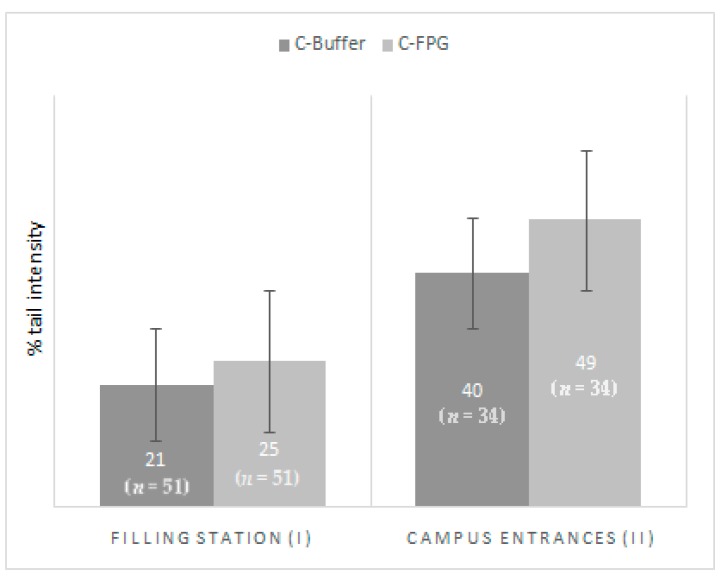
Result of the comet assay with FPG for the two groups of workers exposed to benzene. C-Buffer: mean of samples treated with buffer; C-FPG: mean of samples treated with buffer + FPG enzyme.

The biomarkers of genotoxicity of the workers from the two groups are also shown in Table 4, except for the results of the comet assay with FPG (C-FPG), which are shown in Figure 4. The four variables of chromosomal alteration presented non-normal distribution, but the variables MCA and NCBk could be log-transformed. Differences between the mean values of NCBk, Frag, SPC, BNC, and BEC were encountered between the Group I and Group II workers (Table 4). When the results of the chromosomal alterations for the different categories of tobacco use were compared, differences were found between the workers who had never smoked and those who were smokers, the means being 34 ± 36 and 82 ± 132 for MCA, 2 ± 3 and 6 ± 12 for NCBk, 3 ± 5 and 11 ± 22 for Frag, and 1 ± 1 and 3 ± 4 para SPC, respectively. As for alcohol use, no differences were found between the different categories. The same comparison was done for MN, BEC, and BNC and no difference was found.

The Spearman correlation yielded correlations between MCA and GST (R = −0.368, *p* = 0.0001), total leukocyte count (R = +0.292, *p* = 0.006), and neutrophil count (R = +0.237, *p* = 0.028). It also yielded a positive correlation between SPC and THIOL (R = +0.244, *p* = 0.023). However, when the analyses were controlled by sex, age, tobacco and alcohol use, correlations between MCA and SOD activity (R = +0.373, *p* = 0.015), and between THIOL and the biomarkers MCA (R = +0.409, *p* = 0.007), NCBk (R = +0.330, *p* = 0.033), Frag (R = +0.352, *p* = 0.022), and SPC (R = +0.426, *p* = 0.005) were observed.

Correlations were also identified (by Spearman) between MN and neutrophils (R = +0.400, *p* = 0.0001); BEC and the variables RBCs (R = −0.246, *p* = 0.022), hemoglobin (R = −0.243, *p* = 0.024), hematocrit (R = −0.353, *p* = 0.001), and basophils (R = −0.434, *p* = 0.0001), as well as between BNC and RBCs (R = −0.312, *p* = 0.004), hematocrit (R = −0.364, *p* = 0.0001), and basophils (R = −0.404, *p* = 0.0001). As for the enzymes of oxidative stress, positive correlations (using Spearman) were found between THIOL and the biomarkers BEC (R = +0.268, *p* = 0.012) and BNC (R = +0.217, *p* = 0.044), while negative correlations were found between MDA and the biomarkers BEC (R = −0.341, *p* = 0.002) and BNC (R = −0.236, *p* = 0.037). When the analyses were controlled by sex, age, tobacco and alcohol use, correlations between BEC and the variables basophils (R = −0.271, *p* = 0.034) and monocytes (R = −0.282, *p* = 0.028), as well as between BNC and the variables RBCs (R = −0.281, *p* = 0.028) and basophils (R = −0.312, *p* = 0.014) were observed.

Concerning the genotoxicity biomarkers evaluated, chromosomal aberrations and nuclear abnormalities are more frequently used [11]. To our knowledge, this is the first time C-FPG and methylation of *KLF6* gene have been tested for the biomonitoring of exposed populations. However, due to a series of experimental problems, the evaluation of methylation was not successful and data from only nine filling station workers were obtained, not enough to make any inferences. The methylation averages of LINE-1, *CDKN2B*, and *KLF6* for the nine workers were 67.7% (0.6), 16.1% (10.6), and 0.8% (0.6), respectively.

The C-FPG results did not yield any statistically significant differences between the workers from Group I and Group II. When the results for the different categories of tobacco use were compared, no differences were found, and the same applies to the different categories of alcohol use. As for the clinical data evaluated, the Spearman correlation yielded negative correlations between C-FPG and RBCs (R = −0.285, *p* = 0.008), hematocrits (R = −0.335, *p* = 0.002), neutrophils (R = −0.370, *p* = 0.0001), and lymphocytes (R = −0.233, *p* = 0.032); and between C-FPG and the biomarkers of oxidative stress GST (R = −0.279, *p* = 0.010) and MDA (R = −0.367, *p* = 0.001). These correlations did not remain when controlled by sex, age, tobacco and alcohol use.

The associations between the biomarkers of oxidative stress and genotoxicity and the variables age, duration of exposure, and exposure group are shown in Table 5. The variable “length of time in current job” yielded positive correlations with C-FPG (R = +0.269, *p* = 0.017) and MCA (R = +0.368, *p* = 0.001), and negative correlations with GST (R = −0.246, *p* = 0.028) and MDA (R = −0.421, *p* = 0.0001). However, when controlled by sex, age, tobacco and alcohol use, these correlations disappeared.

Multiple linear regression analyses were performed using the final outcome variables MCA, MN, and C-FPG as dependent variables. MCA showed a significant multiple linear relationship (F(13.499,30.989) = 2.296, *p* = 0.037, R^2^ = 0.285) with the predictor variables of age, duration of exposure, *S*-PMA, CAT, GST, THIOL, MDA, and C-FPG, and the model was significant for CAT (ß = −0.323, t = −2.407, *p* = 0.020), GST (ß = −0.364, t = −2.620, *p* = 0.012), and THIOL (ß = +0.289, t = +2.071, *p* = 0.044). A significant multiple linear relationship was also found for MCA [F(13.838,39.093) = 2.825, *p* = 0.011, R^2^ = 0.303] when *S*-PMA was replaced with *tt*MA, with the model remaining significant for CAT (ß = −0.266, t = −2.081, *p* = 0.042), GST (ß = −0.561, t = −3.659, *p* = 0.001), and THIOL (ß = +0.469, t = +3.279, *p* = 0.002).

The variables age and duration of exposure were used as controls. The variables sex, alcohol use, tobacco use, and SOD did not influence the models obtained for different dependent variables and were not used in the last regression model. When the linear regression model was performed with all the variables, it lost its power of statistical significance.

The multiple linear relationship for C-FPG was significant [F(515,1769) = 3.432, *p* = 0.0005, R^2^ = 0.338], with the predictor variables being age, duration of exposure, *S*-PMA, CAT, GST, THIOL, and MDA; the model was significant for GST (ß = −0.292, t = −2.328, *p* = 0.024) and THIOL (ß = −0.321, t = −2.583, *p* = 0.013). The multiple linear relationship for C-FPG with *tt*MA instead of *S*-PMA was not significant.

**Figure 5:**
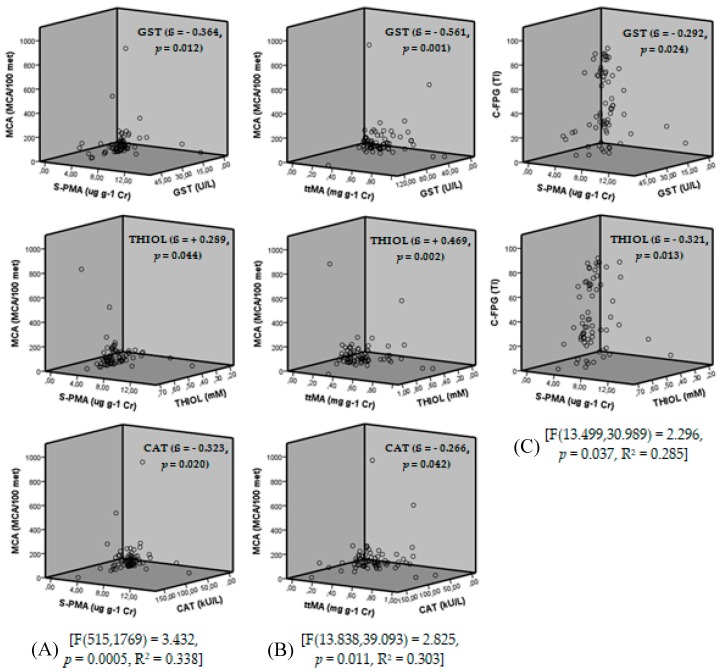
Plot of linearity between biomarkers of exposure, oxidative stress, and genotoxicity. (**A**) Multiple linear regression using MCA as a dependent variable and *S*-PMA, CAT, GST, THIOL, MDA, and C-FPG as predictor variables; (**B**) Multiple linear regression using MCA as a dependent variable and *tt*MA, CAT, GST, THIOL, MDA, and C-FPG as predictor variables; (**C**) Multiple linear regression using C-FPG as a dependent variable and *S*-PMA, CAT, GST, THIOL, MDA, and MCA as predictor variables. All the models were controlled by age and duration of exposure. The variables sex, alcohol use, tobacco use, and SOD did not influence the models obtained for the different dependent variables and were not used in the last model of regression.

When used as a dependent variable, MN did not yield any statistically significant model. The variables sex, alcohol use, tobacco use, and SOD did not influence the models obtained for the different dependent variables. The plot of linearity between biomarkers of exposure, oxidative stress, and genotoxicity can be seen in Figure 5.

## 4. Discussion

Benzene exposure from fossil fuels such as gasoline is a major concern for environmental, occupational, and health entities in Brazil and worldwide, due to the risk it poses to human health [1,2,3,4,5]. The results of the quantification of benzene and toluene in atmospheric air at both the workplaces evaluated (filling stations and campus entrances) showed concentrations of less than 0.01 ppm, confirming exposure to low concentrations of these compounds. Despite the low concentration of benzene in the atmospheric air, this does not exclude the risk of illness for these workers, because there is no safe limit for exposure to carcinogens, as is the case of benzene [4]. Furthermore, there are no studies that prove the safety of occupational or environmental exposure to toluene [5]. Indeed, studies have shown that even low environmental concentrations of these compounds may cause a number of harmful effects on health, like sperm anomalies, fetal growth restriction, cardiovascular diseases, and respiratory dysfunction, while also indicating their potential action as endocrine disruptors [51]. As such, even at exposure levels of less than 0.01 ppm, the possibility of a risk of illness for both groups of workers analyzed should not be ruled out.

In Brazil, there are not enough studies on occupational exposure in filling station workers to meet the needs of these workers and adequately characterize the risks they face, especially when related to biological monitoring. According to Moura-Correa et al. [52], occupational exposure at filling stations affects more than 184,733 workers at 39,450 filling stations throughout the country. To date, few studies on the genotoxicity assessment of workers’ exposure at Brazilian filling stations have been published [12,13,14,15,16,19,20]. In addition to the exposure evaluations at Brazilian filling stations, a recent review was carried out to assess the use of genotoxicity biomarkers for these types of occupational studies between 1995 and 2015 [11]. The data obtained for the evaluations conducted in our study do not differ from other data from Brazil or elsewhere except in comparison to the higher benzene content of fuels in the 1990s.

Italy is one of the few countries where exposure assessments and biomonitoring have been carried out at filling stations, such as the study by Lagorio et al. [53], who determined individual benzene exposure levels (8h TWA) at 1.73 mg m^−3^ (around 0.5 ppm) in Rome. In the early 2000s, higher benzene levels (0.37 ppm, i.e., 1.2 mg m^−3^) were reported at filling stations in Rio de Janeiro [54]. Carrieri et al. [10] also identified environmental benzene concentrations ranging from 11–187 μg m^−3^ at filling stations in northern Italy (values were considerably reduced after the 1990s, to below 0.05 ppm). In Hyderabad (India), environmental benzene and toluene concentrations at filling stations have been measured at 1322 μg m^−3^ and 696 μg m^−3^, respectively [55]. In Bangkok (Thailand), benzene and toluene concentrations in ambient air during work shifts from workers at filling stations have been measured at 107.68 ppb (374 μg m^−3^) and 226.68 ppb (788 μg m^−3^), respectively, while the concentrations in ambient air samples taken from the roadside in front of the six evaluated filling stations were determined as 68.38 ppb (238 μg m^−3^) for benzene and 147.05 ppb (511 μg m^−3^) for toluene [2].

Rosa et al. [16] performed BTEX determinations at three filling stations in the city of Porto Alegre, southern Brazil, finding that benzene was not detected and toluene ranged from 0.2 to 1.5 ppm (638–4792 μg m^−3^). Our research group has assessed BTEX levels at filling stations in different parts of Rio de Janeiro. In the city’s north zone, BTEX determinations in air samples from five different filling stations ranged from 6.4 to 492.5 μg m^−3^ for benzene (with peaks of about 1200 μg m^−3^ associated with tank truck unloading processes) and 7.5 to 110.5 μg m^−3^ for toluene. Compared to the average benzene concentration at filling stations in the west zone of Rio de Janeiro (around 15 μg m^−3^ benzene, displaying more homogeneous data than the north zone), concentrations were 30 times higher in some north zone stations due to their location between high-rise buildings, which confine the benzene, which has a higher density than air. This was not observed in the west zone filling stations on multi-lane highways, which are open and not hemmed in by the surroundings buildings [20].

Importantly, evaluations in outdoor settings may underestimate the levels of exposure these individuals have in indoor environments. In a study by Bolden, Kwiatkowski & Colborn [51], the levels of benzene, toluene, ethylbenzene, and xylenes (BTEX) measured in indoor settings were 16 times higher than the concentrations measured out of doors. This means that the general public is also exposed environmentally to these compounds, especially if they live in the vicinity of certain industrial facilities and/or filling stations. The risk of exposure is caused by the spread of these vapors to the areas surrounding the establishment in different directions and concentrations, according to the direction of the wind and local features [56].

According to Carrieri et al. [10], the general population is subject to environmental exposure because of the volatilization of the solvents present in gasoline emitted both at filling stations, when storage tanks and vehicles are filled, and in vehicle emissions, resulting in widespread diffusion. This environmental exposure can be seen by observing the benzene/toluene emission ratio (B/T). The B/T ratio tends to be used to ascertain the origin of these compounds in the atmosphere, with benzene being attributed to vehicle emissions and toluene to both vehicle emissions and evaporation processes. B/T emission ratios were calculated for the Group I and Group II workplaces, giving the values of 0.74 and 0.23, respectively. According to Martins et al. [57], when the B/T ratio is between 0.2 and 0.5, it tends to indicate a predominance of vehicle emissions, as is the situation of the Group II workers (who work at the campus entrances). Values of over 0.5 indicate the presence of sources of benzene emissions [57], which is typical of the work activities of the Group I workers (filling station employees), who handle a direct source of benzene.

In this study, the mean benzene concentration in the atmospheric air was higher at the Group I than the Group II workplaces, but this difference was not observed for toluene. When the benzene and toluene concentrations in atmospheric air were observed by percentile, an increase in the benzene and toluene concentrations at the 75th percentile could be seen (Table 2), with a higher level at the filling stations (Group I). Furthermore, some of the samples taken at both sites—filling stations and campus entrances—gave values below the limit of quantification, indicating variations in the toluene and benzene concentrations in the sampling. The variation was found to be greater at the filling stations (Group I). These results indicate oscillations in the chronic exposure to which workers are submitted during the working day, which were different for the two groups.

These oscillations in concentrations indicate a limitation to the extrapolation of the results of the environmental evaluation to all the workers from the same type of workplace. Acknowledging this, the evaluation of individual exposure of these workers by determining the biomarkers of exposure *tt*MA and *S*-PMA was considered to give a better idea of their real exposure levels. The *tt*MA and *S*-PMA results demonstrate similar exposure to benzene in the workers from both groups. Although Group I workers were exposed to higher concentrations of benzene in their work activities, this subtle difference was not great enough to be picked up by the biomarkers of exposure *tt*MA and *S*-PMA. This likely occurred because human exposure to low concentrations of benzene in atmospheric air yields urinary metabolite profiles with 70–85% phenol, 5–10% hydroquinone, 5–10% *tt*MA and catechol, and less than 1% *S*-PMA [58,59].The low concentrations of *tt*MA and *S*-PMA excreted in urine probably contributed to the absence of any differentiation in exposure of the workers from the two groups with chronic exposure to benzene, while possibly also hampering the observation of any significant correlation between the two biomarkers.

A study by Carrieri et al. [60], that evaluated workers at an oil refinery in southern Italy, found *tt*MA and *S*-PMA concentrations of 79.6 ± 110.1 μg g^−1^ Cr and 4.13 ± 7.75 μg g^−1^ Cr, respectively. They also assessed a group of agricultural workers without any occupational exposure who lived in the same area, whose *tt*MA and *S*-PMA results were 43.4 ± 48.7 μg g^−1^ Cr and 0.58 ± 1.15 μg g^−1^ Cr, respectively. The *S*-PMA concentrations of the workers from Group I and Group II in our study were lower than those found for the oil refinery workers by Carrieri et al. [60], but higher than the agricultural workers in the same study, constituting an intermediate level of environmental and occupational exposure. However, the *tt*MA values of both groups of workers in Italy were higher than those of the two groups of workers in Brazil in this study, possibly because *tt*MA is influenced by different cultural habits of each country as eating.

The levels of *tt*MA encountered in the urine samples of the workers from Group I and Group II were below the baseline levels reported in the literature, which vary from <0.01 to 0.66 mg g^−1^ Cr [61,62]. Recent studies have investigated the association between urine *tt*MA concentrations and occupational/environmental exposure to benzene, suggesting that *tt*MA is not sensitive enough as a biomarker for monitoring exposure to benzene at concentrations below 0.1 ppm, which is the situation of the study population here [60,63,64]. Melikian et al. [65] did an extensive study of the specificity and sensitivity of the biomarkers of benzene, *tt*MA and *S*-PMA, at low exposure levels and found that the variation in the percentage of benzene transformed into *S*-PMA and *tt*MA diminished as the concentration of benzene itself diminished, especially the conversion of benzene into *tt*MA. Accordingly, Melikian et al. [65] suggest that *S*-PMA is a more sensitive marker of exposure to benzene at low concentrations than *tt*MA.

*tt*MA is not specific only to benzene exposure but may be influenced by the ingestion of potassium sorbate, a food additive present in a great variety of foods. *tt*MA is indicated as a reference for analyses of occupational exposure to benzene of 1 ppm, but at very low concentrations *tt*MA suffers interference from other factors. Thus, even if just 0.12 to 0.18% of sorbic acid is absorbed by the human organism and excreted in urine in the form of *tt*MA, this constitutes a confounding factor for *tt*MA analyses of non-smokers exposed to low concentrations of benzene and with a diet of approximately 500 mg day^−1^ sorbic acid. One way of getting round this limitation could be to evaluate this biomarker before and at the end of the working day [64,66]. Furthermore, *tt*MA may also suffer metabolic interference when there is concomitant exposure to toluene (mainly the situation of the Group I workers in this study), resulting in reduced excretion of *tt*MA [36,67].

Another possible source of exposure to benzene and another potential confounding factor is smoking, which can also affect the excretion of *tt*MA. Smokers tend to have higher levels of urine *tt*MA than non-smokers. As 20% of Group I and 21% of Group II were smokers, there was no significant interference of smoking in the results for the two groups, corroborating the statement that the benzene exposure of both groups of workers was related to their work activities and the air quality in their workplaces. Another factor that may have contributed to the absence of any significant interference of smoking in the results of the two groups was the fact that smoking is not permitted by any of the workers from either group during their work shifts, especially the workers from Group I, due to explosion risks. It is important to note that the maximum peak concentration related to benzene exposure occurs in a few minutes, with the excretion of about 50% of unchanged benzene occurring through the lungs, and 16% also expelled unchanged by the lungs after distribution throughout the organism. Only 34% of the total absorbed benzene is metabolized, mainly by the hepatic pathway, resulting in urinary metabolite *tt*MA and *S*-PMA profiles lower than 1%. Thus, the quick metabolism of this compound and its low biotransformation into *tt*MA and *S*-PMA may have contributed to the absence of any difference between the smoker and non-smoker groups, especially considering that these workers may have smoked before starting their shifts.

The fact that both groups of workers had similar exposure to benzene likely contributed to the non-identification of differences in the results of the clinical evaluation. Even though there were no significant clinical alterations, a positive correlation was still found between total leukocyte count (R = +0.275, *p* = 0.020) and segmented neutrophils (R = +0.272, *p* = 0.022). In addition, although the mean leukocyte count was within the range of normality for the workers studied in this research, the leukocyte count of 13 workers (15%) was slightly lower than 4500 cells/mm^3^, which was the cutoff point set by our study group for warranting attention by the worker, since even if this does not yet represent an actual adverse outcome, it still indicates that these workers’ health should be followed up closely [49]. It is generally accepted that there is some association between chronic exposure to benzene and hematological alterations, and that these alterations may affect both RBCs, white blood cells, and platelets [49]. The literature also associates exposure to the compounds in fuel vapors, benzene included, with a lower total leukocyte count, with the potential for the development of leucopenia [68].

Incidentally, it is important to note that acute myeloid leukemia is one of a heterogeneous group of clonal diseases of hematopoietic tissue, which is characterized initially by the abnormal proliferation of myeloid progenitor cells (myeloblasts), causing insufficient production of normal mature blood cells, with consequent replacement of normal tissue [69]. Over time, this replacement of normal hematopoietic tissue from the marrow leads to neutropenia, anemia, and platelet disease. Because of this, one seemingly contradictory result was that a linear relationship was found between the leukocyte count (F(2,66) = 2.970, *p* = 0.058, R^2^ = 0.083) and the predictor variable *tt*MA (ß = 5169, t = 2.204, *p* = 0.031), but no similar relationship was found when *tt*MA was replaced by*S*-PMA, probably because of the low values encountered.

Although both groups of workers have chronic exposure to low levels of benzene, the oscillations in the exposure of the Group I workers, because of certain labor activities they engage that expose them to higher levels of benzene, should not be ignored. These activities include refueling cars, filling the underground fuel tanks, doing fuel quality tests, and cleaning the filling stations [4]. These are all times when the workers are exposed to higher concentrations of benzene, probably increasing the risk of effects on their health and potential to get sick.

The biomarkers of oxidative stress used in this study, THIOL and MDA, proved to be sensitive to the subtle difference in exposure between the two groups of workers, with the mean THIOL level being lower and the mean MDA level being higher for Group I. Correlations were found between the biomarkers of oxidative stress, indicating that increased GST activity, related to benzene exposure, is associated with increased MDA concentrations (R = +0.369, *p* = 0.001). This is probably because GST is a phase II drug metabolizing enzyme that initially acts in the conjugation of glutathione (GSH) with the benzene molecule, by bonding with the thiol groups present in the amino acid cysteine, while other enzymes, like CAT and SOD, act by degrading reactive oxygen species (ROS). Meanwhile, MDA is a product of lipid peroxidation, indicating the effect of the reaction of ROS with cell membranes [11,15]. Higher GST activity prompted by benzene exposure could help reduce the concentration of the reactive metabolites from benzene, while also protecting cells and genetic material from possible damage, and is consequently related to a reduction in the number of thiol groups available, since these are conjugated with the benzene molecule. Meanwhile, higher MDA levels are related to increased levels of lipid peroxidation in cells, resulting from exposure to benzene [11,15].

An analysis of plasma thiol groups indicates the number of plasma sulfhydryl groups associated with proteins, which are susceptible to oxidative damage. It is important to understand that the analysis of thiol groups is an analysis that is directly associated with GSH levels, since GSH is an important source of thiol groups and other proteins, like albumin, which is the most abundant protein in human plasma and a powerful extracellular antioxidant [41]. For this reason, one of the most important mechanisms related to cellular response to chemical exposure is the glutathione/glutathione *S*-transferase (GSH/GST) system. The response mechanism via the GSH/GST system involves alterations in GSH levels and/or expression of the genes that codify the enzymes involved in GSH synthesis, alterations in the transport of GSH conjugates (increased efficiency of the transport of conjugates with increased GSH and/or increased expression of genes that codify the transporters of these conjugates), and/or alterations in the expression of the genes that codify GST [70]. In fact, the expression of the genes that codify GST is found to be higher in tumor cells than in non-tumor cells. It has also been demonstrated that GSH depletion may increase cell sensitivity to alkylating agents, observed in chemotherapy drugs. These results underline the importance of the GSH/GST system in the cell’s response to chemicals [70].

As for damage to genetic material, in this study differences were found between the chromosomal anomalies NCBk, Frag, and PCS, and the nuclear anomalies BEC and BNC between the two groups of workers exposed to benzene, but no differences were found in the MN or C-FPG tests. One potential explanation for this could be that both groups of workers had similar exposure to benzene, as demonstrated by the *tt*MA and *S*-PMA determinations.

Studies have used research of nuclear anomalies in buccal mucosal cells to investigate the genotoxicity of occupational and environmental exposure to fossil fuel products [12,13,71,72]. The MN results of this study were not statistically significant. Goethel et al. [71] had a similar finding in a study of genotoxicity to assess occupational exposure in filling station workers and taxi drivers. However, a study by Sellappa et al. [59] in India showed a strong association between exposure to fossil fuel vapors during the workday and the development of nuclear abnormalities, especially BEC, which is a type of nuclear abnormality used as a biomarker of gene amplification in genotoxicity studies. Although it is a less widely applied biomarker than the micronucleus of peripheral blood lymphocytes, some authors have assessed benzene exposure in fuel stations through buccal mucosal micronuclei, such as Çelik et al. [73], Benites et al. [12], Hallare et al. [74], Sellappa et al. [72], Rosa et al. [16], and Lacerda et al. [13]. This method is easy to implement, has lower capital investment requirements, and is associated to precision obtained by scoring higher amounts of cells [11]. The data obtained in the present study for buccal mucosal micronuclei from filling station workers in the west zone of Rio de Janeiro are similar to those reported by Çelik et al. [73]. The MN data for buccal mucosal cells obtained by Rosa et al. [16] were higher than those obtained in this study, although both reported low air benzene levels. The other aforementioned studies also reported higher values than this study, with no environmental benzene measurements obtained for comparison [12,13,72,74]. It should be emphasized that the cited studies were carried out in different countries and conditions, reflecting heterogeneous realities [11].

As for C-FPG, a possible explanation for the lack of difference between the results of the workers from the two groups could have to do with the fact that the test was done on frozen whole blood samples, making them less sensitive than results from samples with isolated lymphocytes. Once recent genomic lesions are processed by the DNA repair enzymes, they may potentially be corrected and not form DNA breaks, and thus not be observed in the C-FPG test [75]. Even so, as a dependent variable, C-FPG yielded a significant multiple linear relationship [F(515,1769) = 3.432, *p* = 0.0005, R^2^ = 0.338] with GST (ß = −0.292, t = −2.328, *p* = 0.024) and THIOL (ß = −0.321, t = −2.583, *p* = 0.013), and had the following predictor variables: age, duration of exposure, *S*-PMA, CAT, GST, THIOL, and MDA. In other words, 33% of the appearance of damage to the genetic material assessed by C-FPG was related to reduced GST activity and reduced thiol groups, indicative of the relationship between genotoxicity and oxidative stress.

As a dependent variable, MCA also presented a significant multiple linear relationship [F(13.499,30.989) = 2.296, *p* = 0.037, R^2^ = 0.285] with the variables CAT (ß = −0.323, t = −2.407, *p* = 0.020), GST (ß = −0.364, t = −2.620, *p* = 0.012), and THIOL (ß = +0.289, t = +2.071, *p* = 0.044), with the predictor variables being age, duration of exposure, *S*-PMA, CAT, GST, THIOL, MDA, and C-FPG. This result was maintained [F(13.838,39.093) = 2.825, *p* = 0.011, R^2^ = 0.303] when *S*-PMA was replaced by *tt*MA, when the correlation with CAT (ß = −0.266, t = −2.081, *p* = 0.042), GST (ß = −0.561, t = −3.659, *p* = 0.001), and THIOL (ß = +0.469, t = +3.279, *p* = 0.002) was maintained. This shows that approximately 30% of the alterations observed in the MCA results could be explained by oxidative stress, which is closely related to the detoxification of the organism—i.e., exposure to toxic compounds, such as benzene.

It is therefore important to stress that in this study, even though the results of *tt*MA and *S*-PMA did not differ across the two groups of workers, the chromosomal anomalies (NCBk, Frag, SPC) and nuclear anomalies (BEC and BNC) were major, sensitive biomarkers for the small difference in exposure to benzene identified in the environmental assessment to determine benzene in the atmospheric air. Likewise, the biomarkers C-FPG and MCA were important final outcomes that characterize the relationship between genotoxicity and oxidative stress.

As for chromosomal anomalies, the Group I workers had 4.8 and 4.5 times more NCBk and Frag than the Group II workers. The higher level for Group I (filling station workers) indicates a higher risk of developing an actual adverse outcome due to their occupational and environmental exposure. However, PCS was slightly higher for Group II, which may be why no difference in chromosomal anomalies was observed, which corresponds to all the types of alterations evaluated (NCBk, Frag, and SPC). The results for BEC and BNC were also higher for the Group II workers. One explanation for this—and a limitation of this study—could be the small sample size, which could have resulted in a random variation in the results of the two groups of workers analyzed. It is also important to remember that both the population groups have similar exposure to benzene, as identified by the biomarkers *tt*MA and *S*-PMA.

Any increase in chromosomal and nuclear anomalies indicates a stress factor level higher than the individual’s genetic repair capability. For this reason, MCA, NCBk, Frag, SPC, MN, BEC, and BNC are important biomarkers of cytotoxic effects when the aim is to prevent a disease process, since the increased frequency of C-FPG does not itself constitute an actual adverse outcome for an individual [11]. Santos-Mello and Cavalcante [76] published one of the first Brazilian articles to assess chromosomal aberrations in filling station attendants, finding a correlation between exposure and the occurrence of chromosomal deletions in 49 attendants, which was 11 times higher than in the 24 subjects from the control group.

By observing sister chromatid exchange and chromosomal aberrations, Çelik & Akbaş [77] assessed the effect of the occupational exposure to fuel vapors of 30 filling station assistants as compared with 30 individuals without occupational exposure, both of which groups had 15 smokers, finding that exposure in the workplace together with the habit of smoking was responsible for a significant increase in the occurrences of chromosomal aberrations and sister chromatid exchange, providing evidence for the effect of exposure on the hematopoietic system. This would explain why the workers who were smokers had higher frequencies of MCA than the non-smokers. However, Fracasso et al. [78], Lovreglio et al. [79] and Trevisan et al. [18] found that the MCA test was not sensitive enough to identify effects relating to workers’ exposure to low levels of benzene from fuel vapors.

MCA also correlated negatively with GST (R = −0.368, *p* = 0.0001) and positively with total leukocyte count (R = +0.292, *p* = 0.006) and neutrophil count (R = +0.237, *p* = 0.028). THIOL correlated positively with MCA (R = +0.409, *p* = 0.007), NCBk (R = +0.330, *p* = 0.033), Frag (R = +0.352, *p* = 0.022), and PCS (R = +0.426, *p* = 0.005). GSH is the main sulfhydryl group detected in the thiol group and is associated to the GSH/GST antioxidant system. As such, any reduced GST activity, even with an increase in the concentration of total sulfhydryl groups expressed by the thiol group, leads to less efficient detoxification and exposes genetic material to toxic xenobiotics, which cause genomic and cytotoxic damage. This explains the negative and positive correlations between genomic and cytotoxic damage, as evaluated by MCA, NCBk, Frag, and SPC, and alterations in leukocyte and neutrophil counts and the biomarkers GST and THIOL, respectively. That is why it is important to emphasize that under normal conditions, the cell levels in thiol groups are related to the mechanism of detoxification via GSH/GST. By the way, GSH plays a central role in the biotransformation and elimination of xenobiotics and in defending cells against oxidative stress, representing a fundamental biological adaptation for the survival and assured perpetuity of many species. This tripeptide is found intracellularly in high concentrations essentially in all aerobic organisms, and is the most abundant low-molecular-mass thiol group [70].

Another important point was the fact that the length of time the workers had been in their present jobs correlated positively with C-FPG (R = +0.269, *p* = 0.017) and MCA (R = +0.368, *p* = 0.001), and negatively with GST (R = −0.246, *p* = 0.028) and MDA (R = −0.421, *p* = 0.0001). As such, the longer the worker had been in the current job (which is directly related to length of occupational exposure), the greater the damage to genetic material, as observed by C-FPG and MCA. However, this longer duration appears to cause the organism to adapt, bringing about a reduction in the activity of GST and MDA, a metabolite of lipid peroxidation. This is why the correlations of THIOL and the enzymes involved in the different metabolic pathways of oxidative stress need to be better elucidated to obtain more information on their involvement in the biological process of disease development.

Studies have indicated a correlation between antioxidant enzyme activity and the exposure of filling station workers to fuel vapors. Rekhadevi et al. [55] and Moro et al. [15] described reduced levels of biomarkers of oxidative stress (GSH, SOD, CAT, and GST) and levels of occupational BTEX in exposed workers. Moro et al. [15] also found a negative correlation between exogenous antioxidant concentration from ascorbic acid and damage to genetic material, as assessed by comet assay. In an evaluation of exposure to BTEX of filling station workers, Costa-Amaral et al. [20] found that GST activity correlated negatively with DNA damage, as assessed by comet assay, and positively with length of time in current job, underlining the hypothesis that GST activity may be influenced by longer exposure times to BTEX. However, studies of exposure to BTEX and GST activity are still few in number and inconclusive. Data in the literature on oxidative stress enzymes have produced controversial results and reported both increases and decreases of activity under different types of exposure, which calls for further research in this area [34,46,72]. Some studies have also revealed that GST activity may be lower at the beginning of exposure and/or during the development of a given disease, but may subsequently return to normal [80,81].

Recent evidence indicates that human beings metabolize benzene most efficiently at low than at high environmental concentrations, due to the saturation of one of the two benzene metabolic pathways, suggesting a higher risk of leukemia at low environmental exposure levels [25]. This would explain the influence of time in current employment on the genotoxicity results observed in our study. Recent genomic damage is processed by DNA repair enzymes and may potentially be reverted in this process [82,83]. However, as an individual spends more time in the same workplace, a buildup of frequency of DNA damage (C-FPG, MCA, BEC, and BNC) can be observed. Likewise, lower activity of the detoxification enzyme GST after a long period of exposure to benzene could contribute to increased concentrations of the reactive metabolites from benzene, thereby ceasing to protect the genetic material from oxidative damage and leading to the appearance of more genetic material damage [82,83].

It is important to highlight that although benzene features as the main compound of toxicological importance in volatile solvents at filling stations, the genomic material damage observed by MCA in this study cannot be attributed exclusively to benzene, given the exposure of these workers to multiple compounds. Furthermore, there are no studies that prove the occupational safety of repeated exposure to BTEX amongst filling station workers. As this was a cross-sectional study and has no follow-up, it suffers the limitation of not giving any further information on the influence of length of time working at a filling station; cohort studies would be needed to shed more light on this issue. Therefore, studies relating benzene exposure, antioxidant enzyme activity, and genotoxicity are limited in number and inconclusive, making more research in this area an imperative.

Regarding the DNA methylation analysis, despite the low number of samples evaluated, the LINE-1 gene results showed average levels similar to those found amongst filling station workers in Italy by Bollati et al. [47] and Fustinoni et al. [84]. The mean levels of methylation found for the *CDKN2B* gene were similar to the maximum values found by Seow et al. [85] for petrochemical workers in Bulgaria, with higher benzene exposure levels. Mean levels of methylation for *KLF6* gene were not found for populations exposed to benzene. However, *KLF6* gene is identified as having a probable epigenetic mechanism involved in the proliferation of many kinds of tumor cells, and in the reduction in neutrophilia of acute myeloid leukemia [86], as it is a known tumor suppressor [87]. In addition, it is important to note that a decrease in *KLF6* expression is associated with benzene exposure [88].

Finally, the use of genotoxicity biomarkers in the assessment of occupational and environmental exposure to benzene together with biomarkers of exposure demonstrated potential for the early identification of exposure-related damage. Although the biomarkers of exposure are chemical-specific, they may not translate the broad toxic potential of these chemicals, because genotoxic and/or carcinogenic compounds may damage genetic material even at very low doses, as is the situation of this study relating exposure to benzene, where there is no safe level of exposure. That is why there is an increasing use of biomarkers of genotoxicity and cytotoxicity, which can assess the damage caused to genetic material, like MCA, MN, and C-FPG.

## 5. Limitations of the Study

The strategy of this study was to assess two populations whose workplaces are on the same busy highway in the highly polluted metropolis of Rio de Janeiro, Brazil, in a bid to assure similar background pollution levels. Toluene levels were also evaluated in order to calculate benzene/toluene ratios and thus better characterize the type of benzene emission each group of workers was exposed to. This strategy allowed us to observe differences in benzene exposure between the two groups, meeting certain conditions discussed by epidemiology experts in situations where there is uncertainty in the comparability of the controls. The selection of workers at campus entrances (Group II), with characteristics similar to those from Group I (i.e., biological parameters, socioeconomic and education factors, similarities in work activities, including working out of doors and on foot, and no smoking at work), was designed to permit inferences to be drawn on the pollution background of the two groups. This strategy has been applied successfully in other studies, such as in the study carried out by Tunsaringkarn et al. [2], who compared benzene and toluene concentrations in the workplace areas of filling stations with values at the roadside in front of each filling station.

Despite this finding concerning general data comparisons, one significant difficulty hampering more direct comparisons is the scarcity of published articles and limited number of countries where filling stations still have attendants. Self-service pumps are now common in most developed countries, where the majority of monitoring studies are carried out [11]. Other aspects also add to this difficulty, such as the very limited number of papers presenting assessments of BTEX concentrations in the air around filling station pumps.

We attempted to assay new biomarkers of genotoxicity (C-FPG and methilation of *KLF6*) in the biomonitoring of populations exposed to benzene; however, this was not possible due to limitations on the type of study and problems with experimental techniques. To our knowledge, neither C-FPG nor methylation of *KLF6* have ever been cited before in evaluations of populations exposed to benzene.

Another point is that this was a cross-sectional epidemiological study. The determination of causality is one of the limitations of this kind of approach, since the exposure and the effect are assessed at the same time. Thus, without a temporal evaluation, it cannot be confirmed whether the increase in the leukocyte count is associated with changes to the hematopoietic system. Also, individuals who fall sick will be absent from work and therefore less likely to be included in a cross-sectional study.

Similarly, it cannot be affirmed that the increase in the oxidative and genotoxic effect associated with the increased levels of the exposure biomarkers *tt*MA and *S*-PMA corresponds to the development of disease in the workers evaluated, such as acute myeloid leukemia. In addition, the results found could not be compared with results from workers with higher levels of exposure. Thus, the supralinear dose-response relationship for benzene metabolism at low doses could not be verified.

However, although the mean leukocyte count and the increase in the oxidative and genotoxic effects of the workers from both groups was not outside the range of normality, the risk of illness or even effects on the health of these workers cannot be ruled out.

## 6. Conclusions

In this study, benzene and toluene concentrations in atmospheric air were lower than 0.01 ppm at the workplaces of both groups of workers. However, this does not rule out the risk of the development of diseases related to this exposure. The results also demonstrate a greater oscillation in the concentration of benzene in the atmospheric air at the Group I workplaces (filling stations), whose workers are exposed to higher concentrations of benzene in their work activities, which include handling direct sources of benzene. However, *tt*MA and *S*-PMA were not sensitive to this oscillation in concentrations, indicating a similar level of exposure to benzene for both groups of workers. The evaluation of exposure through the biomarkers *tt*MA and *S*-PMA was considered to be more representative of the real concentrations of benzene that these workers were exposed to than the environmental assessment, due to the individualization of the exposure analyses undertaken.

In the clinical evaluation, no difference was found between the two groups of workers exposed to benzene. Even so, 15% of all the workers had leukocyte counts of slightly less than 4.5 × 10^9^ cells L^−1^, warranting the worker’s attention and demanding close medical follow-up.

The biomarkers of oxidative stress (THIOL and MDA), chromosomal anomalies (NCBk, Frag, SPC), and nuclear anomalies (BEC and BNC) were sensitive to the small difference in benzene exposure identified in the evaluation of benzene in atmospheric air, even though the results for *tt*MA and *S*-PMA did not differ between the two groups of workers. The biomarkers C-FPG and MCA were important final outcomes, characterizing a correlation between genotoxicity and oxidative stress through multiple linear relationship. The higher frequency of MCA in Group I indicates an increased risk of an actual adverse outcome caused by occupational exposure in which a direct source of benzene is handled. However, this does not mean that the Group II workers also exposed to benzene would not be at risk of illness for the same reason.

These results show how close a relationship there is between exposure to benzene, even at low concentrations, and its possible effects related to oxidative stress and damage to genetic material. Furthermore, a significant correlation between length of time in current job and the biomarkers C-FPG, MCA, GST, and MDA are related to the genotoxic and cytotoxic effects brought about by exposure to benzene. C-FPG and methylation of *KLF6* were firstly tested as potential genotoxicity biomarkers for the evaluation of benzene populations exposed to benzene, but the results obtained in this work were not significant.

Both of the groups of workers studied were exposed to low concentrations of benzene, which itself already constitutes an increased risk of illness. Therefore, studies that include the biological monitoring of populations exposed to low concentrations should continue to be conducted, as should studies to elucidate the mechanisms of action of benzene together with other hydrocarbons and their effects on health.

## Figures and Tables

**Table 1:** Sociodemographic variables and risk factors of the two groups of workers exposed to low concentrations of benzene.

Variables	Groups of Workers Exposed to Benzene				
	Filling Stations (I)		Campus Entrances (II)		Total	
	*n* (*n* = 51) *	%	n (*n* = 35) *	%	*n* (*n* = 86) *
**Age group (years)**					
≥18 to <25	11	21.6	2	5.7	13
≥25 to ≤39	26	51.0	8	22.9	34
>40	14	27.5	25	71.4	39
**Sex**					
Male	43	84.3	29	82.9	72
Female	8	15.7	6	17.1	14
**Race/ethnicity**					
White	14	29.2	4	12.5	18
Black/brown	33	68.7	28	87.5	61
Indigenous	1	2.1	-	-	1
Marital status					
Married/separated	33	64.7	25	86.2	58
Single	18	33.3	4	13.8	22
**Education**					
9 years of schooling	15	29.4	6	20.7	21
12 years of schooling	33	64.7	22	75.9	55
Higher ed. (unfinished)	3	5.9	1	3.5	4
**Tobacco use**					
Smokers	10	20.00	6	21.4	16
Used to smoke	9	18.0	6	21.4	15
Never smoked	31	62.0	16	57.1	47
**Alcohol use**					
Drinks	36	70.6	14	50.0	50
Used to drink	3	5.9	3	10.7	6
Never drank	12	23.5	11	39.3	23

* n = number of individuals (subtotals differ because of individuals with lost data); SD = standard deviation.

**Table 2:** Results of the environmental and biological evaluation of exposure of the two groups of workers exposed to benzene.

	Group of Workers Exposed to Benzene										
Variables	Filling Stations (I)					Campus Entrances (II)					
	*n*	Mean (SD)	Median	25th Percentile	75th Percentile	*n*	Mean (SD)	Median	25th Percentile	75th Percentile	*p*-Value
Benzene (µg m^−3^)	30	14.85(9.85)	12.96	≤LQ	21.46	18	≤LQ (-)	≤LQ	≤LQ	≤LQ	0.0001*^†^
Toluene (µg m^−3^)	30	20.18 (14.30)	17.03	≤LQ	27.04	18	24.19 (67.80)	6.70	≤LQ	10.04	0.008^†^
*tt*MA (mg g^−1^ Cr)	51	0.24 (0.19)	0.18	0.13	0.30	35	0.26 (0.17)	0.23	0.12	0.32	0.392
*S*-PMA (µg g^−1^ Cr)	36	3.54 (2.47)	≤LQ	≤LQ	4.45	32	3.38 (0.76)	≤LQ	≤LQ	3.34	0.301

SD = standard deviation; *n* = total number of samples collected; Cr = creatinine; LQ = limit of quantification; *p* = *p*-value, [*^†^ *p* < 0.05], with * referring to the statistical difference of the means between the workplaces of the two groups of workers (I and II); and ^†^ referring to the statistical difference of the medians between the workplaces of the two groups of workers (I and II).

**Table 3:** Results of biomarkers of exposure to benzene and of the variable tobacco use in both groups of workers.

	Biomarkers of Exposure									
Tobacco Use	*tt*MA (mg g^−1^ Cr)					*S*-PMA (µg g^−1^ Cr)				
	*n*	Mean (SD)	Median	Minimum	Maximum	*n*	Mean (SD)	Median	Minimum	Maximum
**Filling Stations (I)**										
Never smoked	23	0.19* (0.19)	0.14	<LQ	1.00	23	3.58 (2.54)	<LQ	<LQ	14.60
Used to smoke	6	0.17 (0.07)	0.13	<LQ	0.27	6	<LQ	<LQ	<LQ	<LQ
Smokers	5	0.45* (0.26)	0.50	0.13	0.82	5	3.43 (0.57)	<LQ	<LQ	4.45
**Campus Entrances (II)**										
Never smoked	8	0.27 (0.19)	0.21	<LQ	0.56	8	3.60 (1.18)	<LQ	<LQ	6.52
Used to smoke	3	0.18 (0.06)	0.15	0.15	0.25	3	<LQ	<LQ	<LQ	<LQ
Smokers	4	0.24 (0.10)	0.26	<LQ	0.32	4	3.24 (0.12)	<LQ	<LQ	3.41

SD = standard deviation; *n* = total number of samples collected; LQ = limit of quantification; *p* = *p*-value, [* *p* < 0.05], with * referring to the statistical difference of the means between “never smoked” and “smokers”.

**Table 4:** Results of the biomarkers of oxidative stress and chromosomal and nuclear anomalies of the two groups of workers exposed to benzene.

	Group of Workers Exposed to Benzene								
Variables	Filling Stations (I)				Campus Entrances (II)				
	*n*	Mean (SD)	Minimum	Maximum	*n*	Mean (SD)	Minimum	Maximum	*p*-Value
**Oxidative stress**									
CAT (kU/L)	50	41.37 (32.91)	5.62	154.65	28	37.06 (13.47)	11.65	87.56	0.468
GST (U/L)	50	20.23 (17.97)	1.98	121.25	28	13.47(4.12)	0.00	20.08	0.169
SOD (U/mL)	50	1.90 (1.14)	0.08	5.50	28	1.87 (0.97)	0.11	4.21	0.851
THIOL (mM)	50	0.40 (0.13)	0.19	0.98	28	0.44 (0.05)	0.32	0.55	0.001*
MDA (µM)	50	5.39 (5.53)	0.56	24.51	28	1.19 (0.54)	0.66	2.70	0.0001*
**Chromosomal aberrations**									
MCA (MCA/100 met)	51	73.25 (132.04)	0	841	35	72.43 (100.21)	2	570	0.474
NCBk (NCBk/100 met)	51	6.80 (11.49)	0	73	35	1.40 (2.53)	0	13	0.0001*
Frag (Frag/100 met)	51	11.90 (22.03)	0	141	35	2.80 (5.07)	0	26	0.002*
PCS (PCS/100 met)	51	2.18 (4.37)	0	27	35	2.51 (2.61)	0	9	0.046*
**Nuclear Abnormalities**									
MN (MN/1000 cells)	51	1.19 (1.28)	0	6.00	35	0.72 (0.51)	0	1.50	0.237
BEC (BEC/1000 cells)	51	0.39 (0.74)	0	2.50	35	2.14 (1.47)	0	5.50	0.0001*
BNC (BNC/1000 cells)	51	0.12 (0.28)	0	1.50	35	0.91 (0.90)	0	3.50	0.0001*

SD = standard deviation; met: metaphases; *p* = *p*-value, [* *p* < 0.05], with * referring to the statistical difference of the means between the biomarkers between the two groups of workers (I and II) exposed to benzene.

**Table 5:** Results of the association of biomarkers of oxidative stress, chromosomal aberrations, nuclear abnormalities, and comet assay with FPG per age group, sex, length of time in current job, tobacco use, and exposure group.

**(a) Oxidative stress biomarkers per age group, sex, length of time in current job, tobacco use, and exposure group**											
	**Oxidative Stress**										
	***n***	**CAT (kU/L)**		**GST (U/L)**		**SOD (U/mL)**		**THIOL (mM)**		**MDA (µM)**	
**Variables**	**(*n* = 86)**	**Mean (SD)**	**CI 95%**	**Mean (SD)**	**CI 95%**	**Mean (SD)**	**CI 95%**	**Mean (SD)**	**CI 95%**	**Mean (SD)**	**CI 95%**
**Age group (years)**											
≥ 18 a≤25	13	28.21 (13.12)	19.88–36.55	20.20 (10.58)	13.47–26.92	1.55 (0.90)	0.98–2.13	0.41 (0.12)	0.34–0.48	9.01 (7.77)	3.79–14.23
>25 a ≤39	34	49.68 (37.42)	36.19–63.18	19.50 (20.39)	12.15–26.85	2.01 (1.32)	1.53–2.48	0.43 (0.14)	0.38–0.49	4.77 (4.79)	2.98–6.56
≥40	39	37.28 (16.80)	31.83–42.72	14.89 (8.69)	12.07–17.70	1.97 (0.92)	1.67–2.27	0.41 (0.06)	0.39–0.43	1.70 (1.28)	1.27–2.13
**Sex**											
Male	72	41.19 (25.09)	35.29–47.08	16.82 (14.80)	13.34–20.30	1.94 (1.02)	1.70–2.18	0.43 (0.10)	0.40–0.45	3.60 (4.70)	2.44–4.75
Female	14	39.09 (35.76)	18.44–59.73	19.89 (11.91)	13.02–26.77	1.80 (1.35)	0.98–2.61	0.39 (0.11)	0.33–0.46	5.34 (5.43)	2.06–8.62
**Length of time in the current job (years)**											
<7	45	40.33 (31.52)	30.87–49.80	20.25 (18.57)	14.58–25.73	1.85 (1.20)	1.48–2.21	0.42 (0.13)	0.38–0.46	5.77 (5.94)	3.91–7.62
7–34	35	41.33 (22.44)	33.62–49.04	13.99 (6.71)	11.68–16.29	2.05 (0.95)	1.72–2.37	0.42 (0.06)	0.40–0.44	1.79 (1.24)	1.33–2.23
**Tobacco use**											
Never smoked	47	38.89 (26.07)	31.24–46.55	15.68 (7.89)	13.36–18.00	1.82 (1.06)	1.50–2.13	0.41 (0.09)	0.38–0.44	4.20 (5.28)	2.60–5.81
Used to smoke	15	41.72 (22.44)	29.30–54.14	15.01 (6.31)	11.51–18.50	1.99 (1.03)	1.42–2.56	0.40 (0.07)	0.37–0.44	3.40 (4.44)	0.84=5.97
Smokers	16	44.75 (35.31)	25.93–63.56	26.44 (28.33)	11.34–41.54	2.19 (1.21)	1.55–2.83	0.45 (0.17)	0.36–0.54	3.83 (4.16)	1.54–6.14
**Groups of workers exposed to benzene**											
Filling Stations (I)	51	42.00 (32.89)	32.75–51.25	20.04 (17.84)	15.03–25.06	1.90 (1.12)	1.58–2.22	0.45 (0.05)	0.43–0.47	5.39 (5.53)	3.82–6.96
Campus Entrances (II)	35	39.16(14.46)	34.20–44.13	13.35 (4.44)	11.83–14.88	1.95 (0.99)	1.60–2.29	0.40 (0.13)	0.37–0.44	1.29 (0.73)	1.01–1.56
**(b) Chromosomal aberrations per age group, sex, length of time in current job, tobacco use, and exposure group**											
	**Chromosomal Aberrations**										
	***n***	**MCA (%)**		**NCBk (%)**		**Frag (%)**		**PCS (%)**			
**Variables**	**(*n* = 86)**	**Mean (SD)**	**CI 95%**	**Mean (SD)**	**CI 95%**	**Mean (SD)**	**CI 95%**	**Mean (SD)**	**CI 95%**		
**Age group (years)**											
≥ 18 a≤25	13	49.17 (51.71)	16.31–82.02	3.42 (4.46)	0.58–6.25	5.33 (7.24)	0.73–9.93	1.92 (2.47)	0.35–3.48		
>25 a ≤39	34	69.00 (148.82)	15.35–122.65	6.59 (13.54)	1.71–11.48	12.09 (26.21)	2.65–21.54	2.28 (4.96)	0.49–4.07		
≥40	39	85.08 (111.98)	48.78–121.38	3.67 (5.83)	1.78–5.56	6.46 (10.42)	3.08–9.84	2.46 (3.02)	1.48–3.44		
**Sex**											
Male	72	79.68 (128.31)	49.53–109.83	5.18 (10,06)	2.82–7.54	9.44 (19.18)	4.94–13.95	2.31 (3.90)	1.39–3.22		
Female	14	38.14 (43.08)	13.27–63.02	1.64 (2,67)	0.10–3.19	1.79 (2.36)	0.42–3.14	2.36 (2.95)	0.65–4.06		
**Length of time in the current job (years)**											
<7	45	62.82 (137.69)	21.46–104.19	5.56 (12.00)	1.95–9.16	9.82 (22.91)	2.94–16.70	2.24 (4.46)	0.90–3.59		
7–34	35	89.09 (102.25)	53.96–124.21	3.97 (5.31)	2.15–5.79	7.11 (9.94)	3.70–10.53	2.23 (2.89)	1.24–3.22		
**Tobacco use**											
Never smoked	47	81.91 (132.34)	43.06–120.77	6.28 (11.73)	2.83–9.72	11.47 (22.47)	4.87–18.	2.55 (4.39)	1.26–3.84		
Used to smoke	15	34.20 (36.31)	14.09–54.31	1.67 (2.90)	0.06–3.27	2.67 (4.84)	0.01–5.34	0.67 (0.976)	0.13–1.21		
Smokers	16	48.13 (62.59)	14.77–81.48	4.06 (6.37)	0.67–7.46	6.50 (11.51)	0.37–12.64	2.44 (3.22)	0.72–4.16		
**Groups of workers exposed to benzene**											
Filling Stations (I)	51	73.25 (132.04)	36.12–110.39	6.80 (11.49)	3.57–10.04	11.90 (22.03)	5.70–18.10	2.18 (4.37)	0.95–3.41		
Campus Entrances (II)	35	72.43 (100.21)	38.00–106.85	1.4 (2.53)	0.53–2.27	2.8 (5.07)	1.06–4.54	2.51 (2.62)	1.61–3.41		
**(c) Nuclear abnormalities and comet assay with FPG per age group, sex, length of time in current job, tobacco use, and exposure group**											
		**Nuclear Abnormalities**						**Comet Assay**			
	***n***	**MN (MN/1000 cell)**		**BEC (BEC/1000 cell)**		**BNC (BNC/1000 cell)**		**C-Buffer (TI)**		**C-FPG (TI)**	
**Variables**	**(*n* = 86)**	**Mean (SD)**	**CI 95%**	**Mean (SD)**	**CI 95%**	**Mean (SD)**	**CI 95%**	**Mean (SD)**	**CI 95%**	**Mean (SD)**	**CI 95%**
**Age group (years)**											
≥ 18 a≤25	13	1.13 (1.46)	0.20–2.05	0.96 (1.50)	0.01–1.91	0.33 (0.69)	–0.10–0.77	20.47 (16.56)	9.95–30.99	29.78 (19.94)	17.11–42.46
>25 a ≤39	34	0.97 (1.16)	0.55–1.39	0.66 (1.06)	0.27–1.04	0.23 (0.52)	0.05–0.42	17.88 (17.42)	11.60–24.16	26.22 (23.33)	17.81–34.64
≥40	39	1.01 (0.84)	0.74–1.28	1.45 (1.50)	0.96–1.93	0.60 (0.81)	0.34–0.87	29.46 (20.32)	22.87–36.05	39.83 (27.09)	30.93–48.73
**Sex**											
Male	72	0.96 (1.00)	0.72–1.19	1.06 (1.41)	0.73–1.39	0.44 (0.72)	0.28–0.61	24.30 (20.00)	19.60–28.99	33.60 (26.40)	27.34–39.84
Female	14	1.25 (1.33)	0.48–2.02	1.32 (1.32)	0.56–2.09	0.43 (0.76)	–0.01–0.87	24.62 (17.68)	14.40–34.83	35.53 (22.26)	22.67–48.38
**Length of time in the current job (years)**											
<7	45	1.18 (1.28)	0.79–1.56	0.84 (1.25)	0.50–1.22	0.28 (0.59)	0.10–0.45	18.60 (19.10)	12.86–24.34	25.42 (21.92)	18.76–32.08
7–34	35	0.86 (0.73)	0.61–1.12	1.27 (1.50)	0.76–1.79	0.57 (0.76)	0.31–0.83	29.03 (17.63)	22.98–35.09	41.55 (26.97)	32.29–50.82
**Tobacco use**											
Never smoked	47	1.10 (1.16)	0.75–1.44	1.11 (1.43)	0.69–1.52	0.40 (0.64)	0.22–0.59	21.75 (19.55)	16.01–27.49	32.03 (26.12)	24.36–39.70
Used to smoke	15	0.97 (0.90)	0.47–1.46	0.90 (1.33)	0.17–1.63	0.40 (0.63)	0.05–0.75	35.36 (21.85)	23.27–47.46	46.41 (26.33)	31.22–61.62
Smokers	16	0.75 (0.86)	0.29–1.21	0.81 (0.96)	0.30–1.3	0.34 (0.68)	0.02–0.70	24.59 (17.96)	15.03–34.15	32.92 (25.97)	19.09–46.76
**Groups of workers exposed to benzene**											
Filling Stations (I)	51	1.20 (1.28)	0.84–1.56)	0.39 (0.74)	0.18–0.60	0.12 (0.28)	0.04–0.20	21.34 (20.32)	15.63–27.06	29.52 (24.68)	22.58–36.46
Campus Entrances (II)	35	0.73 (0.51)	0.56–0.90	2.14 (1.47)	1.64–2.65	0.91 (0.90)	0.61–1.22	28.73 (17.72)	22.64–34.81	40.49 (26.04)	31.40–49.58

SD = standard deviation; CI= Confidence Interval (95%).
